# Changes in tear volume and ocular symptoms of patients receiving oral anticancer drug S-1

**DOI:** 10.1186/s40780-018-0100-8

**Published:** 2018-02-07

**Authors:** Reiko Kuriki, Tsuyoshi Hata, Kinuyo Nakayama, Yuichi Ito, Kazunari Misawa, Seiji Ito, Michiko Tatematsu, Norio Kaneda

**Affiliations:** 1 Graduate School of Pharmacy, Meijo University, 150 Yagotoyama, Tempaku, Nagoya, 468-8503 Japan; 2 Department of Pharmacy, Tokai Hospital, 1-1-1 Chiyodabashi, Chikusa, Nagoya, 464-8512 Japan; 3 Department of Nursing, Aichi Cancer Center Hospital, 1-1 Kanokoden, Chikusa, Nagoya, 464-8681 Japan; 4 Department of Gastroenterological Surgery, Aichi Cancer Center Hospital, 1-1 Kanokoden, Chikusa, Nagoya, 464-8681 Japan; 5 Department of Operation, Aichi Cancer Center Hospital, 1-1 Kanokoden, Chikusa, Nagoya, 464-8681 Japan; 6 Department of Pharmacy, Aichi Cancer Center Hospital, 1-1 Kanokoden, Chikusa, Nagoya, 464-8681 Japan

**Keywords:** Tegafur/Gimeracil/potassium oxonate (S-1), 5-Fluorouacil (5-FU), Watering eyes, Lacrimal duct obstruction, Eye disorders

## Abstract

**Background:**

Most eye disorders are not fatal but may deteriorate the quality of life of a patient. The eye disorder that is most frequently reported in the cancer chemotherapy is associated with the combination of tegafur/gimeracil/potassium oxonate (S-1). However, preventive methods or treatment methods for the eye disorder have not yet been established. This study aimed to determine changes in tear volume and subjective ocular symptoms during the treatment period in patients receiving S-1 monotherapy for early detection of adverse effects in the eye and establishment of its treatment methods.

**Methods:**

This study included eleven patients receiving S-1 monotherapy as a postoperative adjuvant chemotherapy for gastric cancer. Six subjective ocular symptoms including watering eyes were evaluated and changes in tear volume measured by the Schirmer’s test in patients receiving S-1 during the treatment period. In the present study, the patients were divided into “no watering eyes” (patients not experienced watering eyes) group and “watering eyes” (patients experienced watering eyes even once) group.

**Results:**

Six out of eleven patients developed watering eyes after receiving S-1 monotherapy. Among these, the earliest onset occurred on the 2nd week after oral administration. Watering eyes and eye discharge were highly related in patients having a trouble in daily life due to the decreased QOL. Changes in tear volume in the “watering eyes” group significantly increased compared to the “no watering eyes” group during the treatment period, especially when the patients had no subjective symptom of the increased tear volume.

**Conclusions:**

It is essential to prevent eye disorders including watering eyes as an adverse effect of S-1 administration. The present study recommends that the tear volume should be periodically measured using Schirmer’s test, and the patient should be interviewed regarding the subjective ocular symptoms for the early detection of watering eyes caused by S-1 administration. If the tear volume can not be measured periodically, medical staffs should pay attention to the patient with eye discharge.

## Background

Recently, significant progress has been made in cancer treatment owing to advanced clinical development of anticancer drugs with novel mechanisms of action. Although different types of combinations of anticancer drugs have been developed, several adverse effects have also been reported. Irrelevant adverse effects have been reported, and they are apparently apart from their mechanism of action, because increasing number of patients use anticancer drugs with expended adhibitions [1–3]. Prevention and/or treatment methods have been instructed as guidelines and publications for major adverse effects such as hematologic toxicity and gastrointestinal toxicity. However, screening or treatment methods for certain adverse effects including eye disorders have not been firmly developed because most eye disorders are not fatal but may deteriorate the quality of life (QOL) of patients. Despite having a poor QOL, patients may remain untreated for eye disorders. Patients may not recognize eye symptoms to be adverse effects that are caused by anticancer drugs administered to them. Even if patients visited an eye clinic, the ophthalmologist may diagnose the eye disorder merely due to aging or dry eye. Consequently, the patient’s eye condition may deteriorate further, leading to poor QOL with decreased eyesight.

According to a report by Nakajima et al. [4], only 39.5% of physicians and 33.3% of pharmacists were aware of adverse effect of the eye disorders listed on package inserts of anticancer drugs. The report also stated that physicians and pharmacists primarily experienced eye disorders in patients during treatment with S-1 [4]. S-1 is an oral anticancer drug comprising tegafur (FT), gimeracil (CDHP), and potassium oxoate (OXO) in a molar ratio of 1:0.4:1. S-1 has been widely used to treat various kinds of cancer in clinical practice and is a crucial drug that is particularly used in adjuvant therapy for resected gastric cancer. S-1 has been reported to be associated with many eye disorders in which watering eyes is most frequently reported. Watering eyes is being recognized as one of the significant clinical complications that deteriorate the QOL of patients treated with S-1. However, the exact mechanism of development of watering eyes remains unclear [5–8]. Moreover, prevention and treatment methods for patients with watering eyes have not been established.

Therefore, identification and prevention of watering eyes are essential, and the establishment of a method for detecting patients with a risk of watering eyes is necessary. Although some studies have reported the development of watering eyes in patients receiving S-1 [9–12], to the best of our knowledge, no study has evaluated the tear volume of patients over an extended period of treatment. Our study aimed to evaluate changes in tear volume and ocular symptoms during the treatment course in patients receiving S-1 monotherapy for the early detection of adverse effect in the eye and establishment of its treatment methods.

## Methods

### Patients and investigation period

The study included 15 patients (male = 9 and female = 6) receiving S-1 monotherapy as an adjuvant chemotherapy after gastric cancer surgery at Aichi Cancer Center Hospital. Written informed consent was obtained from all patients. However, four male patients were excluded from the study due to the withdrawal of consent or moving to another hospital.

In general, S-1 monotherapy is defined as a treatment with S-1 alone. The patients were not administered any other anticancer drugs, and dosage included S-1 40 mg/m^2^ twice a day for 4 weeks, followed by drug holidays for 2 weeks. One course of treatment comprised eight repeats of this protocol. However, according to a physician’s decision, some patients received S-1 for 2 weeks, followed by a week off, and in these patients, one course was defined as a treatment that comprised two repeats of this protocol. However, if patients exhibited abnormal clinical laboratory values and/or gastrointestinal symptoms, the dose of S-1 was reduced, and the drug holiday period was extended.

In this study, only outpatients prescribed with S-1 between August 2015 and January 2016 were selected. Patients were followed up until the scheduled eight courses had been completed or until S-1 administration had been discontinued due to clinical reasons of a patient.

### Survey items

The Schirmer’s test was performed by nurses in the ophthalmology department to measure the tear volume of both eyes a day before oral administration of S-1 and on each consultation day. The tear volume was estimated by converting 1 mm of the height of Schirmer’s test paper to 1 μL equivalent of tears [13]. In addition, patients were interviewed regarding the subjective ocular symptoms for the following six parameters: watering eyes, eye discharge, eye pain, flashing lights, foreign body sensation, and reduced visual acuity. The severity of each symptom was assessed on a five-point scale ranging from level 0 to 4 as follows: level 0 = none, level 1 = infrequently, level 2 = occasionally, level 3 = almost always, and level 4 = always. The patients were also asked whether these symptoms interfered with daily life such as watching TV, reading a book, and driving a car. In the present study, the patients were divided into “no watering eyes” group (patients who did not experience watering eyes) and “watering eyes” group (patients who experienced watering eyes even once). Changes in tear volume and subjective ocular symptoms associated with watering eyes were also examined between the two groups.

Relative Dose Intensity (RDI) during the treatment period was calculated using the following equation: RDI = [actual dose (mg)/week]/[standard dose (mg)/week].

### Statistical analysis

Age, height, body weight, body surface area (BSA), and serum creatinine level (Scr) and dose, RDI, and treatment period (Table 3) were examined using the Mann–Whitney U test. Fisher’s exact probability test was used to compare the differences in sex. Each item of tear volume and its changes in watering eyes was examined using the Kruskal–Wallis H test, and significant differences were further analyzed using a multiple comparison test according to the results of the Mann–Whitney U test with Bonferroni’s correction. Correlation between accumulative dose of S-1 and change in the tear volume was examined using the Spearman’s correlation. A two-tailed *p* < 0.05 was considered as statistically significant.

### Ethical permission

The present study was approved as a clinical study in compliance with “the ethical guidelines on research in medical science for human beings” by the Ethics Review Committee of the Aichi Cancer Center and conducted under a sufficient ethical consideration (Receipt number: 2015–1-021). The first patient was registered on August 5, 2015.

## Results

### Patient characteristics

Eleven patients (male = 5 and female = 6) were selected for this study, and the patients’ characteristics were summarized in Table 1. Patients with a past medical history of eye disorders were appropriately treated. Two patients had cataract; one was treated with pirenoxine eye drops. One patient had a white deposition on the surface of the right black eye and was subsequently followed up. Another patient had dry eye and was self-treated with an eye drop containing a sulfa drug.

**Table 1:** Observation period and reason for cessation of therapy

Case	Treatment period (course)	Reason for cessation of therapy
1	4	Originally scheduled
2	8	
3	8	
4	8	
5	4	Changing the treatment
6	2	Severe watering eyes and fatigue
7	8	
8	2	Changing the hospital
9	8	
10	7	Repeated extension of drug holidays
11	6	Repeated extension of drug holidays

There were six patients in “watering eyes” group and five patients in “no watering eyes” group (Table 2), and there was no significant difference in the patient characteristics between the two groups (Table 3).

**Table 2:** Patients’ medical history of eye disorders and subjective ocular symptoms

Case	Medical history of eye disorders	Subjective ocular symptoms					
		Watering eyes	Eye discharge	Eye pain	Flashing lights	Foreign body sensation	Reduced visual acuity
1	White deposit on surface of the right black eye (followed up)		+				
2	Cataract (after surgery)		+				+
3		+*	+*				+
4		+*	+*	+			+*
5		+	+			+	
6	Cataract (with an eye drop containing pirenoxine)	+*	+*	+		+*	+*
7							
8					+*		+*
9							
10	Dry eye (self-treated with an eye drop containing sulfa drug)	+*					
11		+*	+*		+		

*Subjective symptoms affecting daily life

**Table 3:** Patient characteristics and treatment status

			Watering eyes		No watering eyes		*P*
			*n* = 6		*n* = 5		
Age		(year)	70	(44–80)	63	(43–67)	0.297*
Sex		(male/female)	6	(3/3)	5	(3/2)	1.000**
Height		(cm)	156.15	(147.0–178.0)	146.2	(154.3–164.0)	0.535*
Body weight		(Kg)	46.75	(40.0–58.8)	47.6	(31.2–78.4)	0.548*
BSA		(m2)	1.372	(1.29–1.69)	1.404	(1.16–1.79)	0.548*
Scr		(mg/dL)	0.61	(0.43–0.98)	0.6	(0.49–0.80)	0.575*
Dose	Start	(mg)	100	(80–120)	100	(80–120)	0.535*
	Total	(mg)	13,440	(4880–19,790)	13,440	(5820–22,360)	0.548*
Therapeutic intensity	RDI	(%)	82.68	(49.11–99.41)	79.95	(54.34–95.14)	0.522*
Treatment period	Course		7	(2–8)	8	(2–8)	0.382*
	Days		319	(84–350)	350	(91–371)	0.601*

Age, height, body weight, BSA, Scr,dose, therapeutic intensity, and treatment period are indicated as median (range)

Relative Dose Intensity (RDI) = [actual dose (mg)/week]/[standard dose (mg)/week]

*Mann–Whitney *U*-test; **Fisher’s exact probability test

### Treatment status

In the “watering eyes” group, the median S-1 treatment period was 7 courses, 319 days, whereas in the “no watering eyes” group, the median S-1 treatment period was 8 courses, 350 days. The median (range) total dose of S-1 administered to the “watering eyes” group was 13,440 mg (4880–19,790 mg), whereas that administered to the “no watering eyes” group was 13,440 mg (5820–22,360 mg). In the “watering eyes” group, the median (range) of RDI during the treatment period was 82.68% (49.11–99.41%). In the “no watering eyes” group, the median (range) RDI was 79.95% (54.34–95.14%) (Table 3).

### Onset of watering eyes and subjective ocular symptoms

Regarding the date of onset of watering eyes, the earliest onset was observed on the 2nd week (in the 1st course) after oral administration, and the latest onset was observed on 18th week (in the 3rd course). The symptoms of watering eyes interfered with the patient’s daily life on the 2nd week (in the 1st course) in the earliest case after starting oral administration, whereas for the latest case, the symptoms interfered with daily life on the 22th week (in the 4th course). For one patient, watering eyes did not interfere with daily life (Table 4).

**Table 4:** Period until the onset of watering eyes and subjective ocular symptoms in the “watering eyes” group

Case	Onset of watering eyes		Interference with daily life		Subjective ocular symptoms				
	Week	Course	Week	Course	Eye discharge	Eye pain	Flashing lights	Foreign body sensation	Reduced visual acuity
3	2	1	4	1	+				+
4	14	3	22	4	+	+			+
5	6	1	–		+^a^			+^a^	
6	2	1	2	1	+	+		+	+
10^b^	18	3	21	3					
11^b^	4	1	6	1	+		+		

^a^Subjective ocular symptoms developed prior to watering eyes

^b^Patient with repeated extension of drug holiday

In the “watering eyes” group, one patient was not presented with subjective ocular symptoms other than watering eyes during the treatment period. Prior to the onset of watering eyes, another patient was presented with subjective symptoms of eye discharge and foreign body sensation. In the remaining four patients, additional subjective ocular symptoms were presented at the same time as or after symptoms of watering eyes (Table 4).

In the “watering eyes” group, all patients excluding one exhibited eye discharge, which interfered with daily life of four patients. This showed that watering eyes was associated with a subjective ocular symptom of eye discharge. Watering eyes and eye discharge became more relevant when these symptoms were rather serious to the extent of interfering with daily life (Table 2). The number of patients who complained the worst severity of subjective symptoms of watering eyes in the five-point scale was as follows: level 1, 1 patient; level 3, 2 patients; level 4, 3 patients. In the “no watering eyes” group, three patients exhibited some subjective ocular symptoms excluding watering eyes, which interfered with the daily life in one patient of the three (Table 2).

### Tear volume and its changes in watering eyes

**Fig. 1:**
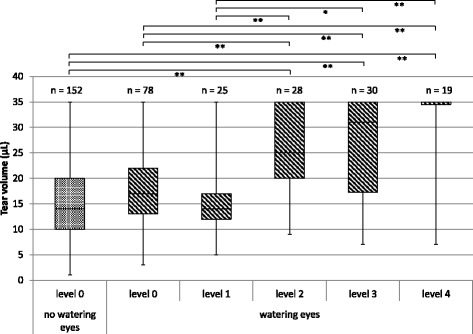
Relationship between watering eyes and the tear volume in the “watering eyes” and “no watering eyes” groups. A five-point scale ranging from level 0–4 is indicated: level 0, none; level 1, infrequently; level 2, occasionally; level 3, almost always; and level 4, always. *P < 0.05; **P < 0.01. The box line, the median value; the upper and lower ends of the box, the third and the first quartile; and the upper and lower ends of the beard, the maximum and minimum values. The median (range) tear volumes (for both eyes) before treatment were 10 μL (3–35 μL) and 17.5 μL (5–35 μL) in the “watering eyes” and “no watering eyes” groups, respectively

When the patients had subjective symptoms of level 0, there was no significant difference in tear volume between the “watering eyes” group and “no watering eyes” group. In the “watering eyes” group, there was no difference in tear volume between patients who had the subjective symptoms of level 0 and level 1. However, the tear volume significantly increased when patients had symptoms worse than level 2, compared with that when patients had symptoms below level 2 (Fig. 1).

**Fig. 2:**
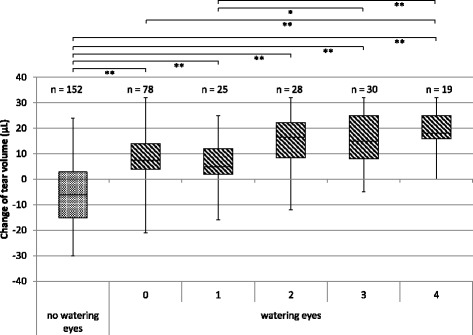
Relationship between watering eyes and a change in the tear volume in the “watering eyes” and “no watering eyes” groups. A five-point scale ranging from level 0–4 is the same as those described in the legend of Fig. 1. *P < 0.05; **P < 0.01. Based on the tear volume at the start of the oral administration of S-1, change in the tear volume was estimated. The box line, the median value; the upper and lower ends of the box, the third and the first quartile; and the upper and lower ends of the beard, the maximum and minimum values

Moreover, for patients who had subjective symptoms of level 0, changes in tear volume significantly increased in the “watering eyes” group compared with that of the “no watering eyes” group. In the “watering eyes” group, changes in tear volume significantly increased as the patient’s symptoms worsened (Fig. 2).

### Correlation between accumulative dose of S-1 and change in the tear volume

**Fig. 3:**
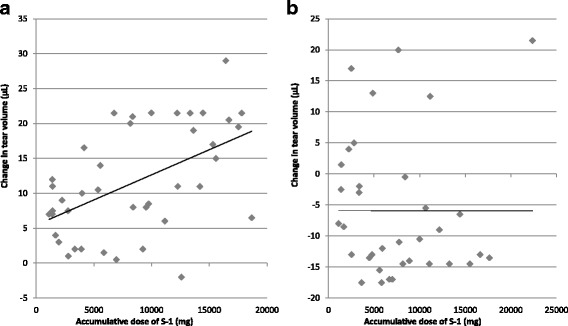
Correlation between the accumulative dose of S-1 and a change in the tear volume. Based on the tear volume at the start of the oral administration of S-1, change in the tear volume was estimated. Tear volume was generally monitored every two weeks during the course of the treatment. Occasionally, data taken two days after the end of a course were included. **a** “Watering eyes” group (y = 0.00072*×* + 5.46; rs = 0.463; r^2^ = 0.264; *P* < 0.01, Spearman’s correlation). **b** “no watering eyes” group (y = − 0.00004*×* – 4.60; rs = 0.197; r^2^ = 0.00045)

The assessment of a correlation between the accumulative dose of S-1 and a change in the tear volume in patients with S-1 monotherapy is imperative. Figure 3a shows a weak but significant correlation between the accumulative dose of S-1 and a change in the tear volume in the “watering eyes” group (correlation coefficient = 0.463). Conversely, no association was observed in the “no watering eyes” group (Fig. 3b).

### Adverse effects other than ocular symptoms

In the “watering eyes” group, while the adverse effects of grade 2 were anemia (2 patients), thrombocytopenia (2 patients), diarrhea (2 patients), and leukopenia (1 patient), an adverse effect of grade 3 was thrombocytopenia (1 patient). In the “no watering eyes” group, the adverse effects of grade 2 were leukopenia (2 patients) and anemia (1 patient).

## Discussion

In the present study, we examined adverse effects in the eye of patients receiving S-1 monotherapy, particularly considering eye disorders such as watering eyes and six subjective ocular symptoms. Schirmer’s test was performed to estimate the tear volume, and changes in both tear volume and the subjective ocular symptoms were investigated. Although the sample size was small, we conducted, for the first time, prospective study and followed through the patients during the period of treatment with S-1. The longest period monitored was over eight courses (about 350 days).

Many adverse effects were assessed according to the Common Terminology Criteria for Adverse Events (CTC-AE) v.4.0; however, three of the six subjective ocular symptoms investigated in the present study, i.e., eye discharge, foreign body sensation, and reduced visual acuity, were not incorporated in the CTC-AE v.4.0. In the CTC-AE v.4.0, the extent of watering eyes is scaled as “grade 1: intervention not indicated,” “grade 2: intervention indicated,” or “grade 3: operative intervention indicated.” Flashing lights is rated as “grade 1: symptomatic but not limiting ADL (activities of daily living),” “grade 2: limiting instrumental ADL,” or “grade 3: limiting self-care ADL.” For the detailed evaluation of the extent of eye disorders, questionnaires regarding subjective ocular symptoms were prepared in this study, wherein patients were asked to provide the frequency and extent of watering.

There were no significant differences between the “watering eyes” group and “no watering eyes” group in the frequency, type, and extent of the subjective ocular symptoms and absolute tear volume before S-1 administration (Fig. 1). It was difficult to identify patients who will suffer from watering eyes before S-1 administration. However, in the “watering eyes” group, it was observed that change in tear volume gradually increased, which may be attributed to the significant changes associated with subjective ocular symptoms even when they were scaled as level 0. In Fig. 2, the data were analyzed using the accumulative number of measurements obtained from a limited number of patients over an extended period of the treatment. In addition, we demonstrated a weak but significant correlation between the accumulative dose of S-1 and a change in the tear volume (Fig. 3a). It might be possible to assess the likelihood of watering eyes if medical staff measures the tear volume in patients before the onset of subjective ocular symptoms. For the treatment or prevention of ocular symptoms caused by the S-1 administration, early detection and rapid treatment are highly recommended [8]. Reportedly, the most effective method for the treatment of watering eyes is to insert a stent into the lacrimal duct [9]. Notably, the utility of eye drops to wash out the surface of the eyes is not validated.

Regarding the onset of watering eyes, we determined that it was based on an individual patient, with the earliest starting from the first course of the treatment. A study on patients with watering eyes who received S-1 monotherapy as adjuvant chemotherapy for gastric cancer in the Shizuoka Cancer Center demonstrated a similar early onset of watering eyes [12].

In this study, watering eyes were observed in 54.5% of patients. According to the package insert of the product, S-1, the incidence rate of watering eyes was 16%, based on the SPIRITS study [14]. In the study of Shizuoka Cancer Center, watering eyes were reported in 25.3% of patients [12]. Perhaps, the higher incidence rate of watering eyes observed in this study than all mentioned above could be attributed to the small sample size of this study and that patients recognized more easily the occurrence of ocular symptoms, such as watering eyes, because they were explained beforehand about the adverse effect.

It is reported that watering eyes was associated with decreased appetite, stomatitis, pigmentation, and rash [12]. In this study, patients in the “watering eyes” group did not experience severe adverse effects, including oral mucositis and rashes, during the first course of S-1 monotherapy. However, these adverse effects were sometimes observed following the onset of watering eyes. Until completing all protocols of S-1 monotherapy, some patients with watering eyes had the similar adverse effect to those reported by Shizuoka Cancer Center. In the “no watering eyes” group, patients were not subjected to a dose reduction of S-1 because they could eat meals tailored to their desired taste several times in a day.

In general, for S-1 monotherapy, physicians assess the adverse effects at 2 or 3 weeks after the start of oral administration; however, patients may have watering eyes before the assessment time. In such patients, it is impossible to identify watering eyes with the above method. Therefore, it is important for medical staff to acquire precise knowledge and provide patients with effective and accurate information to prevent serious adverse effects. The present study recommends that the medical staff should carefully interview the patients on each consultation day regarding the presence of watering eyes. Such interviews should also be performed by community pharmacists through hospital-community pharmacy collaboration system. It may be beneficial because community pharmacists can be involved in a careful interview with a patient [15].

Tears of patients receiving S-1 therapy contain 5-fluorouracil (5-FU), which is the active ingredient of the main metabolite [16–19]. 5-FU excreted into the tear damages corneal epithelial cells and corneal epithelial stem cells, resulting in the onset of corneal disorder and watering eyes [12, 20, 21]. Because gastrointestinal disorders are caused by anticancer drugs owing to epithelial cell damage, they are thought to be associated with watering eyes as well [21]. Thus, when a patient exhibits an increase in tear volume and has gastrointestinal disorders, the patient may be associated with a risk of watering eyes and should be carefully monitored from the early stage. When patients exhibit adverse effects related to gastrointestinal symptoms at an early course, the tear volume must be measured using the Schirmer’s test.

Corneal disorder induced upon intravenously administered 5-FU by instillation has been reported [17]. Another study suggested that corneal disorder is closely associated with the concentration of CDHP in the eyes rather than that of FT or 5-FU [19]. Thus, it is imperative to quantify the concentration of 5-FU and each component of S-1 in tears and analyze their association with eye disorders.

## Conclusions

It is essential to prevent eye disorders including watering eyes as an adverse effect of S-1 administration. However, it is difficult to predict whether a patient will develop watering eyes before receiving S-1 monotherapy. The present study recommends that the tear volume should be periodically measured using Schirmer’s test, and the patient should be interviewed regarding the subjective ocular symptoms for the early detection of watering eyes caused by S-1 administration. If the tear volume can not be periodically measured, medical staff should pay attention to the patient with eye discharge.

## Abbreviations

5-FU: 5-fluorouracil
ADL: Activities of daily living
BSA: Body surface area
CDHP: Gimeracil
CTC-AE: Common terminology criteria for adverse events
FT: Tegafur
OXO: Potassium oxonate
QOL: Quality of life
RDI: Relative dose intensity
S-1: Tegafur/gimeracil/potassium oxonate
Scr: Serum creatinine level
